# One Health in Coastal and Marine Contexts: A Critical Bibliometric Analysis Across Environmental, Animal, and Human Health Dimensions

**DOI:** 10.3390/ijerph22101523

**Published:** 2025-10-04

**Authors:** Alexandra Ioannou, Evmorfia Bataka, Nikolaos Kokosis, Charalambos Billinis, Chrysi Laspidou

**Affiliations:** 1 Civil Engineering Department, University of Thessaly, 38334 Volos, Greece; alexioannou@uth.gr (A.I.); bataka@uth.gr (E.B.); nkokosis@uth.gr (N.K.); 2 Department of Planning and Regional Development, University of Thessaly, 38334 Volos, Greece; 3 Faculty of Veterinary Medicine, University of Thessaly, 43100 Karditsa, Greece; billinis@uth.gr

**Keywords:** planetary health, coastal and marine ecosystems, climate change, zoonotic diseases, integrated health indicators, aquaculture and fisheries, pathogen surveillance, climate–health linkages, cross-sectoral governance, sustainable development goals

## Abstract

Coastal ecosystems sustain biodiversity, food resources, and human livelihoods, yet are increasingly exposed to climate change, pollution, and anthropogenic stressors. These pressures affect not only ecosystem integrity but also human health, highlighting the urgency of adopting the One Health framework. While One Health has gained global prominence, its systematic application in coastal and marine governance remains limited. This study provides the first bibliometric review of One Health research in coastal and marine contexts, analyzing 154 publications from Scopus (2003–2025) using Bibliometrix under PRISMA-S guidelines. Scientific output was minimal until 2015 but accelerated after 2020, peaking at 37 publications in 2024. Less than 20% of studies explicitly integrated all three One Health dimensions. Research has largely centered on environmental monitoring and aquaculture health, with antimicrobial resistance, climate–health linkages, and integrated coastal indicators underexplored. Keyword mapping revealed two distinct yet connected clusters: a biomedical cluster emphasizing antibiotics, resistance, and microbiology, and an environmental cluster focusing on pollution, ecosystems, and zoonotic risks. Outputs are geographically concentrated in high-income countries, particularly the USA, Brazil, and the UK, while contributions from low- and middle-income coastal regions remain scarce. These findings confirm both the rapid growth and the fragmentation of One Health scholarship in coastal contexts. By identifying gaps, trends, and collaboration patterns, this study builds an evidence base for embedding One Health in coastal monitoring, climate adaptation, and governance, advancing multiple United Nations’s Sustainable Development Goals.

## 1. Introduction

The One Health (OH) framework emphasizes the interconnections between human, animal, and environmental health, promoting transdisciplinary approaches to complex challenges [1,2,3,4]. Originally focused on zoonoses and antimicrobial resistance (AMR), OH now extends to planetary health concerns, including climate change and biodiversity loss [5,6,7,8,9].

Coastal zones exemplify the urgency of OH integration: nearly 40% of the world’s population lives within 100 km of the shoreline [10]. These ecosystems provide critical services, food security, disease regulation, and climate adaptation, but are increasingly exposed to climate-driven hazards, urbanization, and pollution [11,12,13,14]. Marine heatwaves and eutrophication trigger harmful algal blooms (HABs) affecting food safety and respiratory health [15,16] warming waters facilitate *Vibrio* spp. outbreaks [17,18] and intensive aquaculture elevates zoonotic and AMR risks [19,20]. These risks intersect with social vulnerabilities, such as weak infrastructure and surveillance [2,6], and are reinforced by land-based pressures including agricultural runoff and deforestation [21,22].

Environmental indicators illustrate OH’s relevance. Chlorophyll-a, widely used in EU directives (WFD, MSFD), links eutrophication with health outcomes [23,24,25,26,27,28]. Microplastics (MPs), often interacting with pollutants, further amplify ecological and health risks [29,30,31]. Yet their integration into OH-based assessments remains limited.

Bibliometric studies show rising OH interest but reveal gaps: biomedical dominance, geographic imbalance, and underrepresentation of climate–health linkages and integrated indicators [32,33,34,35]. Coastal applications are scarce [1,36]. EU directives and global agendas [37,38,39] acknowledge the nexus but rarely operationalize OH in practice.

This study provides the first bibliometric review of OH in coastal and marine contexts, analyzing Scopus-indexed publications (2003–2025) with Bibliometrix under PRISMA-S. By documenting trends, thematic gaps, and collaboration patterns, it builds an evidence base for embedding OH into coastal monitoring and governance, thereby supporting ecosystem-based adaptation and advancing progress toward Sustainable Development Goals (SDGs 3, 6, 12, 13, 14, and 15).

Specifically, the study addresses the following research questions:

1. How has the scientific production on the application of the One Health framework in coastal and marine ecosystems evolved during the period 2003–2025?
2. What are the most frequent thematic domains and research gaps, as revealed by keyword co-occurrence networks and thematic maps?
3. Which sources, authors, and journals contribute most to shaping the field, according to productivity distributions and citation impact?
4. What is the geographical distribution of research, and how are authors, keywords, and journals interconnected in shaping thematic contributions?
5. How is the dual research orientation (biomedical vs. environmental) reflected in the Multiple Correspondence Analysis (MCA)?
6. What is the scope and intensity of international collaborations, and what imbalances appear in the participation of low- and middle-income regions?

## 2. Materials and Methods

We followed a systematic bibliometric process in accordance with the PRISMA/PRISMA-S guidelines to map the literature at the interface of the OH approach with coastal/marine ecosystems. The search was conducted in the Scopus (Elsevier) databases on 11 August 2025 (Europe/Athens, UTC+3), via their native web interfaces. Restricted filters (year and language) were implemented. Specifically, the language was restricted to English and the year, from 2003 to 2025.

### 2.1. Study Design

Guided by PRISMA, we conducted a bibliometric analysis of OH scholarship, examining trends in (a) annual publication output, (b) geographical distribution of publications, (c) leading journals, (d) subject categories, (e) citation impact, and (f) author keywords.

### 2.2. Search Strategy

The search strategy was pre-defined and fully documented (PRISMA-S). For Scopus, the TITLE-ABS-KEY field was used. The common logical framework consisted of: (a) variations/synonyms of the OH approach, (b) vocabulary defining coastal/marine environments, and (c) public health topics relevant to OH in these environments (e.g., zoonoses, antimicrobial resistance, microplastics). Τhe query for Scopus was:

TITLE-ABS-KEY (“one health” OR “integrated health” OR “planetary health”) AND TITLE-ABS-KEY (coast* OR “coastal ecosystem*” OR “marine ecosystem*” OR “coastal area*” OR estuar*) AND TITLE-ABS-KEY (“public health” OR zoonos* OR “environmental health” OR “antimicrobial resistance” OR microplastic*).

The use of the asterisk (*) allowed for right-hand truncation, specifically coast → coastal, coastline. We did not include the gray literature or other sources.

The keyword set was designed to reflect the conceptual breadth of OH and its most policy-salient topics in coastal and marine contexts. Core OH terms (e.g., “one health”, “planetary health”, “integrated health”) have been widely adopted in prior bibliometric mappings to delineate the field’s contours and to capture its interdisciplinary scope across human, animal and environmental dimensions [32,33,34,35]. Environmental descriptors were selected to comprehensively cover coastal and marine settings that feature prominently in governance and assessment frameworks (e.g., MSFD/WFD), hence the use of right-hand truncation for coast and estuary and the inclusion of “marine ecosystem*” [11,12,21,37]. Health-relevant topic terms (e.g., “zoonosis”, “antimicrobial resistance”, “microplastic”) align with OH priorities articulated in global agendas and the scientific literature particularly the Quadripartite OH Joint Plan of Action and WHO’s climate-health framing for vulnerable coastal populations, as well as empirical evidence on AMR and pollutant interactions in aquatic systems [2,29,30,31,32,33,34,35,36,37,38,39,40,41,42].

Regarding possible synonymy, the Boolean logic and truncation were intentionally chosen to include dominant variants used in this literature (e.g., coast → coastal, coastline; estuar → estuary/estuaries*). Less common descriptors (e.g., “littoral”, “shoreline”) are not standard in OH-focused coastal governance/health indexing and were therefore not included. We acknowledge that alternative vocabularies can marginally affect corpus size; however, the adopted terms correspond to prevailing usage in OH bibliometrics and the coastal assessment literature cited above.

### 2.3. Data Collection and Processing

**Figure 1:**
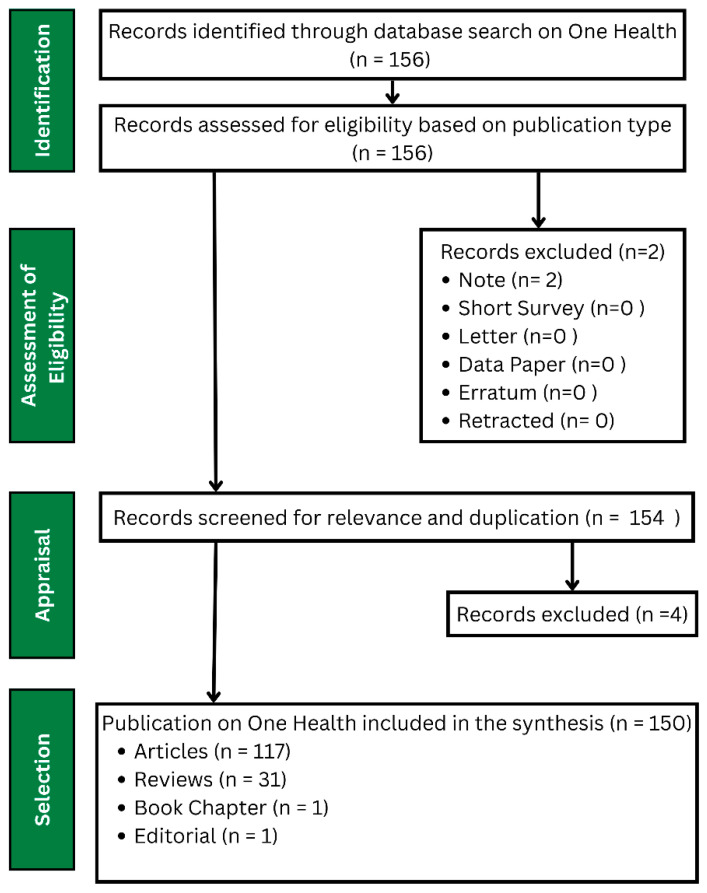
PRISMA flow diagram of the bibliometric analysis. From 156 records retrieved in Scopus (2003–2025), six were excluded, resulting in 150 eligible publications (117 articles, 31 reviews, one book chapter, and one editorial) analyzed under PRISMA-S guidelines.

The range of the years were selected to start from 2003 until 2025, since according to Miao et al. [33], the term “One Health” originated in 2003 by Karesh [43]. However, after the query applied in Scopus the entries started from 2013. Following the inclusion and exclusion criteria, 154 publications were finally obtained (Figure 1). In the Scopus database, the Full Record and Cited References were exported in 1 step and imported into RStudio (v. 2023.12.0.369) using R (v. 4.4.2).

### 2.4. Data Analysis

To conduct the analysis, relevant publications were first retrieved from the Scopus database using carefully defined search queries that matched the research topic, applying appropriate filters for document type, language, and time range. The search results were then exported from Scopus in BibTeX (.bib) format, ensuring that citation information, abstracts, keywords, and references were included in the export settings. The downloaded BibTeX file was subsequently imported into RStudio, the web-and used *bibliometrix* R package [44] (version 5.0.1.9000), which was launched in RStudio (RStudio Desktop 2025.09.0+387 (Build 387) and R version 4.4.2 (2024-10-31 ucrt)).

For the keyword analysis, the following assumptions were applied. The term *animals* combines the words *animals* and *animal*, while *human* combines the words *human*, *male*, *female*, and *adult*. The terms *article* and *review* were excluded from the analysis. The term nonhuman refers to nonhuman species, such as animals, in relation to humans within the context of shared environments, pathogens, and health risks, as described by Esposito et al. [45].

Keyword handling followed established bibliometric practice as implemented in bibliometrix [44]. Specifically, we applied right-hand truncation at the query stage, harmonized frequent lexical variants (e.g., animals/animal, humans, including male, female, adult), excluded generic document-type tokens (e.g., article, review), and retained domain-relevant composite terms (e.g., antimicrobial resistance, HABs, marine environment). The label “nonhuman” is used in the sense common to OH studies, i.e., nonhuman species considered in shared environments, pathogens and risks [45]. These steps ensure comparability with prior OH bibliometrics and reduce artificial fragmentation of closely related terms.

## 3. Results

### 3.1. Analytical Definitions and Bibliometric Laws

To enhance methodological transparency, we briefly outline the bibliometric laws and analytical techniques applied in this study, following their implementation in bibliometrix [44].

Lotka’s Law. Describes the skewed distribution of author productivity, where most authors publish one paper and progressively fewer publish multiple papers. The expected frequency is:

(1) $$f\left(n\right)=\frac{A}{n^{2}}$$

where ***A*** represents the number of single-paper authors and ***f***(***n***) is the number of authors publishing ***n*** papers. 

Bradford’s Law. Explains the concentration of scientific output in a small “core” of journals, followed by zones with exponentially larger numbers of journals but similar total output, typically in a ratio of 1:***n***:***n***^2^.

Keyword co-occurrence. Thematic linkages between keywords were measured with the Association Strength index:

(2) $${AS}_{ij}=\frac{c_{ij}}{\sqrt{c_{i}\cdot c_{j}}}$$

where $c_{ij}$ is the number of co-occurrences, and $c_{i}$, $c_{j}$ their overall frequencies.

Thematic mapping. Keywords were classified by centrality (relevance to the field) and density (degree of development) into four quadrants: motor themes, highly developed but isolated themes, emerging or declining themes, and basic or transversal themes.

MCA reduces high-dimensional categorical data (keywords) into a two-dimensional conceptual space, enabling visualization of clusters and proximities between biomedical and ecological themes.

Together, these methods capture productivity patterns, journal concentration, thematic maturity, and conceptual structures, offering a comprehensive picture of OH research in coastal and marine contexts.

Three-field plots (also known as Sankey-type diagrams) are a bibliometric visualization technique that simultaneously represents relationships across three distinct fields (e.g., authors, keywords, and sources). By illustrating the flows between these dimensions, they provide an intuitive understanding of thematic linkages, research dissemination, and the interconnections between scholarly communities and publication outlets. These diagrams are widely employed to trace research trends and to explore the diffusion of topics within the scientific literature.

### 3.2. Bibliometric Patterns of One Health Research in Coastal and Marine Contexts

The operationalization of the OH framework in coastal resilience is becoming increasingly urgent given the interconnected pressures from climate change, AMR, and ecosystem degradation. OH emphasizes the interdependence of human, animal, and environmental health, promoting transdisciplinary approaches to complex socio-ecological challenges [3,4]. In aquatic systems, AMR proliferation is often linked to intensive aquaculture, effluent discharge, and environmental pollution, with direct implications for both human and animal health [46,47]. Climate-driven changes, such as rising sea surface temperatures and altered salinity patterns, can facilitate the expansion of HABs and *Vibrio* spp., increasing public health risks [18,48]. Coastal monitoring programs, particularly those employing chlorophyll-a as a proxy for eutrophication and primary productivity, are essential for early detection of ecological shifts with potential health impacts [37,49]. However, the integration of such environmental indicators into OH-oriented policy and governance remains limited, underscoring the need for harmonized, cross-sectoral monitoring frameworks.

**Figure 2:**
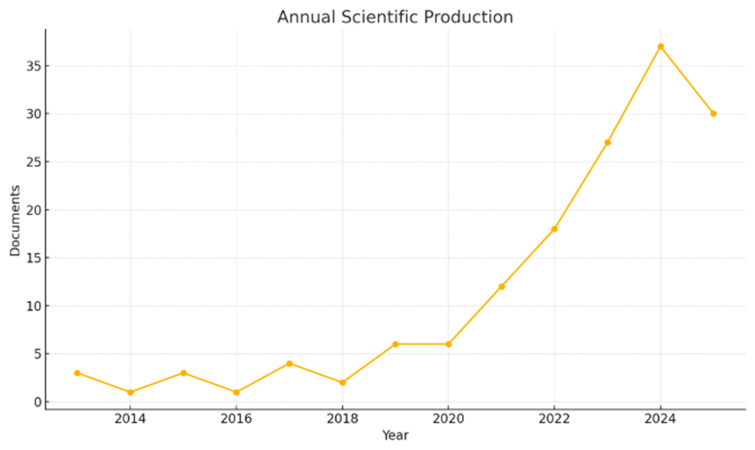
Annual scientific production on One Health in coastal and marine contexts (2013–2025). The output remained low and inconsistent until 2018, then increased sharply after 2020, peaking at 37 publications in 2024 and maintaining high levels in 2025.

According to Figure 2, from 2013 to 2018, annual scientific output was low and inconsistent, with only one to four papers each year. Production steadied at six papers in 2019 and 2020 before accelerating sharply, reaching twelve in 2021, eighteen in 2022, twenty-seven in 2023, and a peak of thirty-seven in 2024. Even the partial 2025 count of thirty papers remains far above earlier levels, underscoring the field’s rapid expansion. Overall, the figure reflects a clear shift after 2020 toward sustained and growing research activity, in line with the rising focus on OH and AMR in coastal and marine contexts [50].

As shown in Table 1, the most cited articles in the field span a diverse range of themes, from foundational frameworks of Planetary and One Health to applied studies on zoonotic diseases, climate change, pollution, aquaculture, and antimicrobial resistance. This thematic diversity illustrates both the conceptual maturity and the applied breadth of the field, complementing the patterns observed in Figure 2. By linking citation impact with the topics addressed, the table provides a more nuanced understanding of the drivers of scientific influence and highlights the domains where further interdisciplinary integration remains necessary.

**Figure 3:**
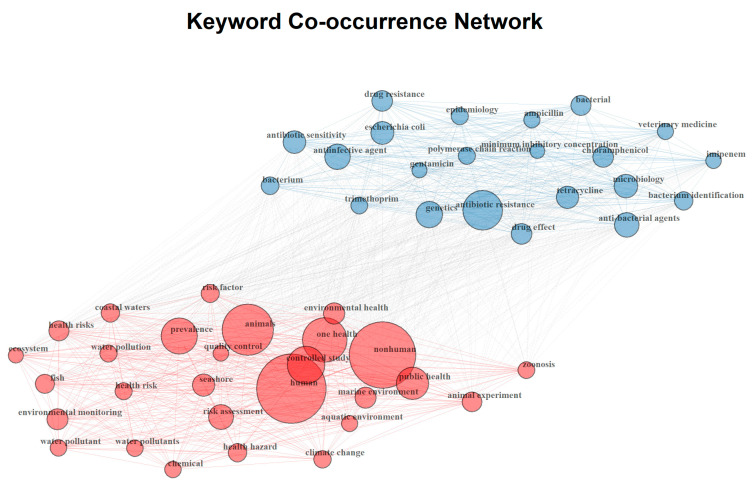
Keyword co-occurrence network of publications related to antibiotic resistance and One Health. Two main clusters are identified: a red cluster emphasizing One Health, public health, environmental health, and zoonosis; and a blue cluster highlighting antibiotic resistance, genetics, and anti-bacterial agents. Node size represents the frequency of keyword occurrence, while link thickness indicates the strength of co-occurrence relationships.

The keyword co-occurrence network (Figure 3) illustrates two main thematic clusters within the literature. The red cluster centers on One Health, human, nonhuman, animals, environmental health, risk assessment, and marine environments, highlighting the interdisciplinary links between human, animal, and ecosystem health in relation to pollution, zoonoses, and public health risks [1,59,60,61,62]. The blue cluster is dominated by antibiotic resistance, microbiology, Escherichia coli, drug resistance, antimicrobial agents, and genetics, reflecting research focused on microbial mechanisms, laboratory methods, and veterinary or medical applications [63,64,65,66]. The size of the nodes indicates the relative frequency of each keyword, while the thickness of connecting lines represents the strength of co-occurrence, showing that although the two clusters are distinct, they are interconnected through shared concerns about antibiotic resistance and its broader OH implications.

**Figure 4:**
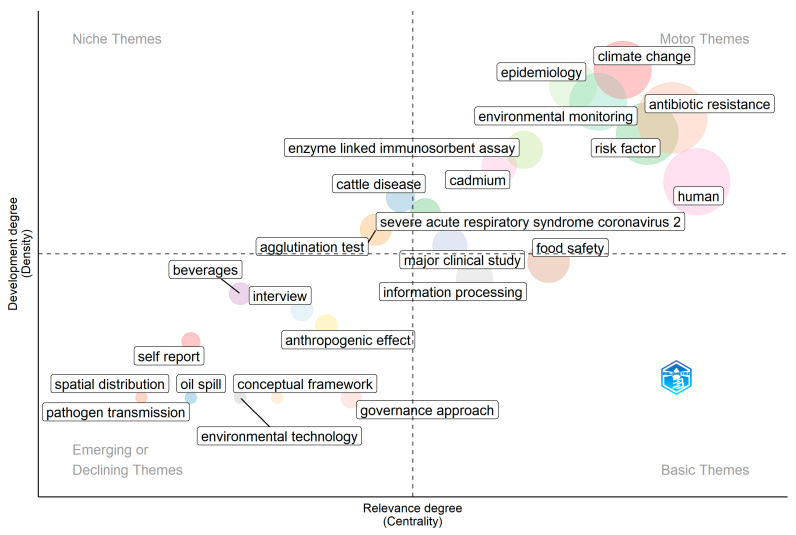
Thematic map of author keywords derived from co-word analysis. Bubbles represent clusters. Axes show centrality (relevance to the field) on the *x*-axis and density (internal development) on the *y*-axis. Motor themes (**upper-right**) include climate change, antibiotic resistance, environmental monitoring, risk factor, human, enzyme-linked immunosorbent assay, and epidemiology. Niche themes (**upper-left**) include cattle disease and agglutination test. Basic/transversal themes (**lower-right**) include information processing and food safety bordering the motor quadrant. Emerging/declining themes (**lower-left**) include governance approach, environmental technology, spatial distribution, oil spill, pathogen transmission, and conceptual framework.

The thematic map (Figure 4) highlights the conceptual structure of the field by positioning topics across four quadrants. Specifically, the motor themes (upper-right quadrant), the niche themes (upper-left quadrant), the basic and transversal themes (lower-right quadrant), the emerging or declining themes (lower-left quadrant). Topics such as climate change, antibiotic resistance, epidemiology, environmental monitoring, risk factors, enzyme-linked immunosorbent assay, and human are presented as motor themes. These are highly relevant and well-developed clusters, indicating that they constitute the core research drivers in the field [2,17,63,67]. Their centrality demonstrates their strong interconnections with other themes, while their density highlights their internal maturity and conceptual robustness. The prominence of climate change and antibiotic resistance reflects the global urgency of these challenges and their cross-cutting influence across multiple disciplines [62,68]. For the niche themes cattle disease, and agglutination test are positioned as niche areas. These clusters are internally well-developed and conceptually specialized, but they exhibit weaker connections to the broader research network. They represent technically advanced yet more peripheral topics that support specific subfields rather than the overall structure [69,70,71]. Clusters such as food safety, major clinical study, and information processing fall into the category of basic and transversal themes. These topics are highly relevant and widely connected but remain less developed. They provide the foundational base upon which other research builds and highlight areas with strong interdisciplinary potential that require further development to reach maturity [1,59,72]. Pathogen transmission, spatial distribution, oil spill, environmental technology, conceptual framework, and self-report are positioned in the quadrant of emerging or declining themes. Their low density and low centrality suggest that they are either nascent topics that could gain momentum in the future or research areas that are losing relevance [68,73]. Distinguishing between emerging and declining trajectories requires complementary temporal analyses of keyword trends [37].

**Figure 5:**
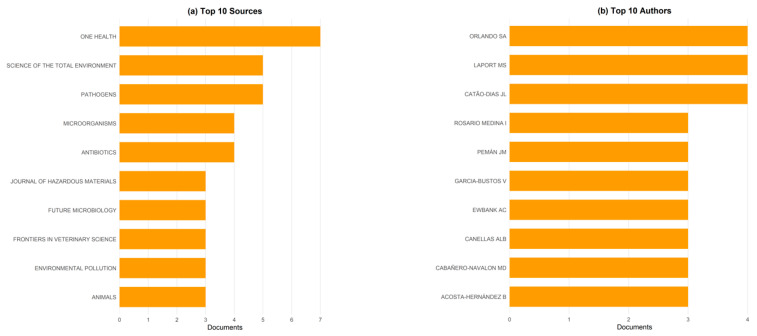
Leading sources and authors in One Health research in coastal and marine contexts (2012–2025). (**a**) Top 10 journals publishing on the topic, with One Health emerging as the most frequent outlet, followed by Science of the Total Environment and Pathogens. (**b**) Top 10 contributing authors, led by Orlando S.A., Laport M.S., and Catão-Dias J.L., each with four publications, followed by a group of researchers with three publications each. The distribution reflects both the concentration of key contributors and the relatively small but active research community driving this field forward.

Figure 5a shows that OH journal is the most frequent source, contributing seven documents, followed by Science of the Total Environment and Pathogens with five each. Microorganisms and Antibiotics each account for four publications, while journals such as Journal of Hazardous Materials, Future Microbiology, Frontiers in Veterinary Science, Environmental Pollution, and Animals contributed three each. This distribution highlights a strong interdisciplinary focus, with top sources spanning environmental sciences, microbiology, veterinary medicine, and public health. The prominence of OH underscores the centrality of integrated human–animal–environment research in this field.

Figure 5b identifies Orlando S.A., Laport M.S., and Catão-Dias J.L. as the most prolific authors between 2012 and 2025, each contributing four publications. They are followed by Rosario Medina I., Pemán J.M., García-Bustos V., Ewbank A.C., Canellas A.L.B., Cabañero-Navalon M.D., and Acosta-Hernández B., each with three documents. The results reflect a relatively small but active group of recurring contributors driving research in this area. Such concentration of authorship suggests strong leadership and expertise, potentially shaping the field’s direction and collaborations.

**Figure 6:**
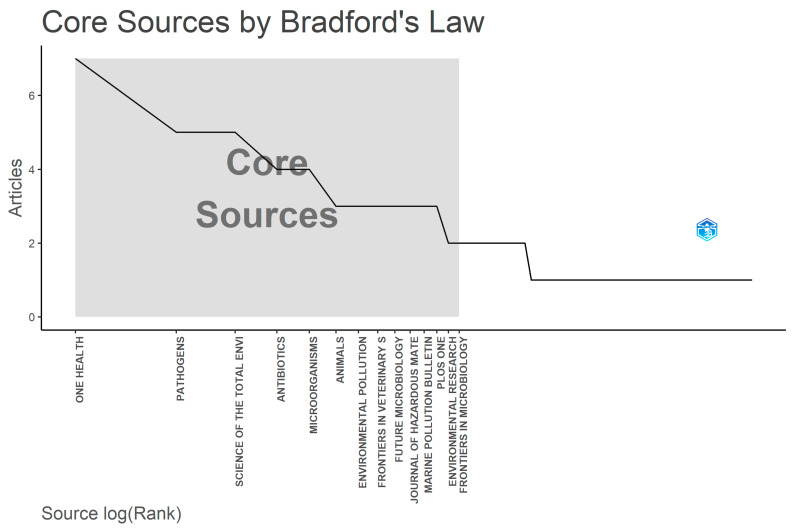
Core sources identified by Bradford’s Law. The plot shows the distribution of articles across journals ranked by productivity. The shaded area highlights the core sources, including One Health, Pathogens, Science of the Total Environment, Antibiotics, and Microorganisms, which contribute the highest concentration of relevant publications in the field.

Figure 6 applies Bradford’s Law to identify the concentration of publications in a limited number of journals central to OH research in coastal and marine contexts. The shaded zone marks the “core sources,” including *One Health*, *Pathogens*, *Science of the Total Environment*, *Antibiotics*, *Microorganisms*, and *Animals*, which together account for the highest density of output. Beyond this cluster, the curve flattens sharply, indicating a long tail of less productive journals that publish only a few articles each. This distribution demonstrates that the field relies on a relatively small set of interdisciplinary journals to anchor its literature base, while the majority of relevant contributions are scattered across diverse outlets, reinforcing both the fragmentation and the specialized focus of research in this domain.

**Figure 7:**
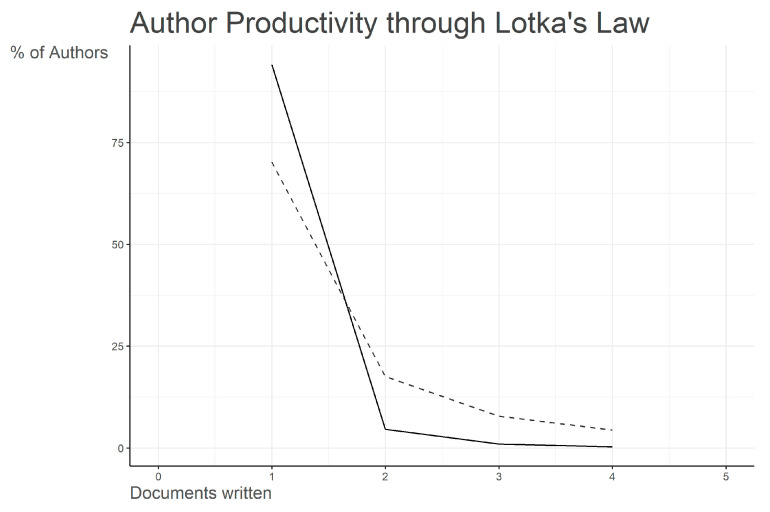
Author productivity distribution according to Lotka’s Law in One Health research on coastal and marine contexts (2013–2025). The majority of authors contributed only a single publication, with steeply declining proportions for those with two or more documents. This distribution reflects a highly skewed authorship pattern, confirming that a small core of recurring scholars sustains much of the output, while most researchers remain occasional contributors. Solid line = Empirical/Observed data (actual author productivity); Dot line = Theoretical Lotka’s Law prediction.

The Lotka’s Law distribution in Figure 7 shows that the overwhelming majority of authors in OH coastal and marine research are one-time contributors, while only a very small minority publish multiple papers. The steep decline from single-paper authors to those with two or more documents reflects a highly skewed productivity pattern, consistent with Lotka’s theoretical model. This confirms that while a handful of recurring scholars provide sustained leadership in the field, most contributions are scattered across occasional authors, underscoring both the emerging nature of this research domain and its reliance on a relatively small core of active researchers.

**Figure 8:**
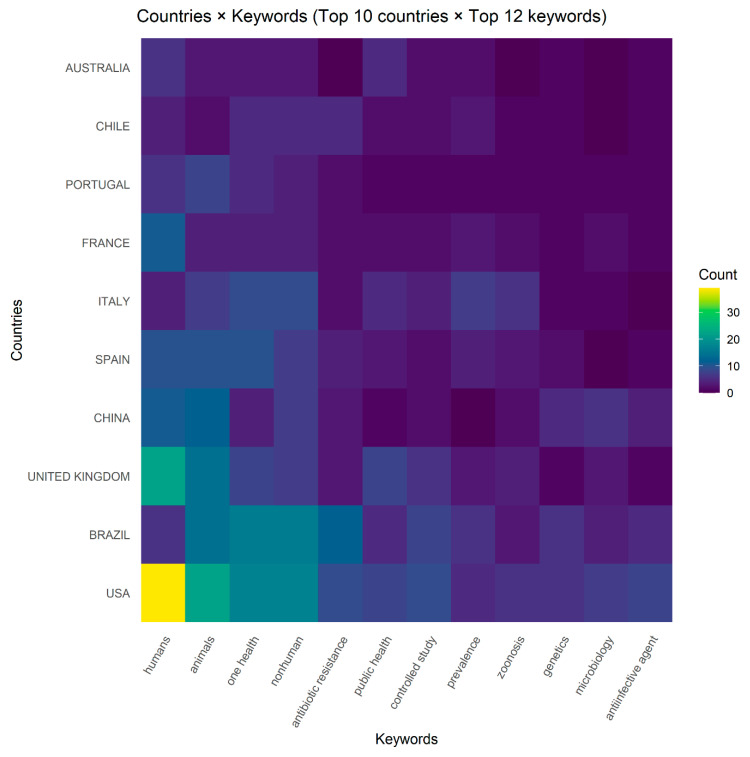
Countries × keywords heatmap (2013–2025). The United States, Brazil, and the United Kingdom show the highest keyword occurrences, with “humans,” “animals,” “One Health,” and “nonhuman” emerging as the most frequent terms, reflecting the dominance of integrative and AMR-related themes in coastal and marine One Health research.

Figure 8 shows that the USA leads in publication volume (153 keyword occurrences), followed by Brazil (101) and the United Kingdom (80). The most frequent keywords are humans (119), animals (96), OH (81), and nonhuman (79), indicating a strong emphasis on integrated human–animal–environment research. Countries such as China, Spain, and Italy contribute substantial outputs, with notable diversity in keyword usage. This distribution aligns with earlier findings that high-income countries dominate OH and AMR research, while middle-income countries play key roles in region-specific studies [33,41,51]. The prominence of *nonhuman* alongside *One Health* reflects growing recognition of cross-species interactions in addressing global health challenges.

**Figure 9:**
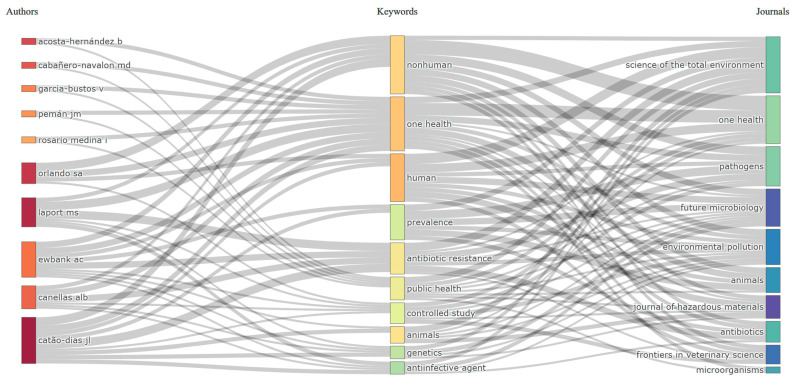
Three-field plot of authors, keywords, and journals. Leading authors link to core terms such as One Health, antibiotic resistance, and public health, which connect to journals including Science of the Total Environment, One Health, and Pathogens. The flows illustrate how researchers shape themes and channel outputs into key interdisciplinary outlets.

Figure 9 presents a three-field Sankey diagram linking countries, keywords, and journals. Brazil and the USA lead contributions, followed by Ecuador, Italy, and the UK, with strong links to core terms such as *nonhuman*, *one health*, *humans*, and *animals*.

Τhe three-fields plot (Figure 9) illustrates the interconnections between authors, keywords, and journals, providing a comprehensive view of how knowledge is produced, thematically structured, and disseminated. On the left, leading authors such as Laport MS, Catão-Dias JL, and Orlando SA show strong links with central keywords, including One Health, human, and antibiotic resistance. This indicates their substantial contribution to shaping the conceptual foundations of the field. At the center, keywords such as nonhuman, One Health, human, antibiotic resistance, public health, and prevalence dominate, reflecting the thematic focus of the literature. The size and positioning of these nodes highlight their recurring use and relevance across multiple studies, reinforcing their role as core topics in the field. On the right, journals such as Science of the Total Environment, One Health, Pathogens, Future Microbiology, and Environmental Pollution appear as the main outlets for disseminating this research. The strong connections between central keywords and these journals demonstrate the interdisciplinary nature of the field, which spans environmental sciences, microbiology, veterinary studies, and public health. Overall, the plot reveals that the conceptual core of the field is structured around One Health and antimicrobial resistance research, with key authors acting as bridges between human- and animal-related studies, and major journals providing the platforms where these interdisciplinary contributions converge.

**Figure 10:**
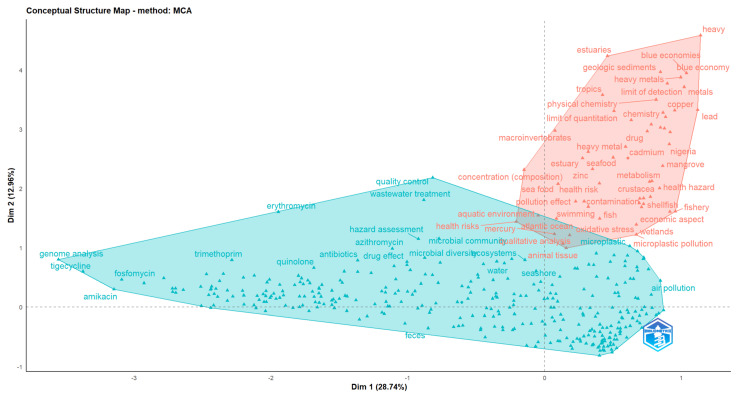
Multiple Correspondence Analysis (MCA) factorial map based on keyword co-occurrence. The first two dimensions explain 41.7% of the total variance (Dim 1: 28.7%, Dim 2: 12.9%). Two main clusters are identified: (i) a biomedical and microbiological cluster on the left, centered on antibiotics, resistance mechanisms, and clinical studies; and (ii) an environmental cluster on the right, associated with heavy metals, microplastics, and ecosystem-related terms. The spatial distribution highlights the dual orientation of the field, bridging clinical/biomedical and environmental/ecotoxicological perspectives within a One Health framework.

The Multiple Correspondence Analysis (MCA) factorial map (Figure 10) illustrates the conceptual structure of the field, based on the co-occurrence of keywords. The first two dimensions explain 41.7% of the total variance (Dim 1: 28.7%, Dim 2: 12.9%), capturing a substantial proportion of the underlying relationships.

Two major clusters are distinguished. On the left (blue cluster), terms such as antibiotics, quinolone, genome analysis, tigecycline, and wastewater treatment appear, reflecting a strong orientation toward clinical, pharmaceutical, and microbiological studies, with emphasis on antimicrobial compounds, resistance mechanisms, and laboratory methods [63,65,66]. On the right (red cluster), keywords including heavy metals, cadmium, lead, copper, microplastic pollution, estuaries, and wetlands dominate, representing an environmental and ecotoxicological perspective, where research focuses on contaminants, ecosystems, and associated health hazards [37].

The spatial separation along Factor 1 highlights the contrast between a biomedical/clinical axis and an environmental/ecological axis, while Factor 2 provides further differentiation between methodological/analytical approaches (bottom) and ecosystem-based assessments (top). The clustering of microplastics, heavy metals, and oxidative stress with ecological terms (e.g., estuaries, mangroves, seafood) underscores the growing convergence of environmental pollution and health risk studies [1].

Overall, the MCA reveals a dual research orientation, where biomedical investigations of antibiotics and resistance co-exist and increasingly intersect with environmental studies on pollutants and ecosystem health, reflecting the integrative OH perspective [59,62].

**Figure 11:**
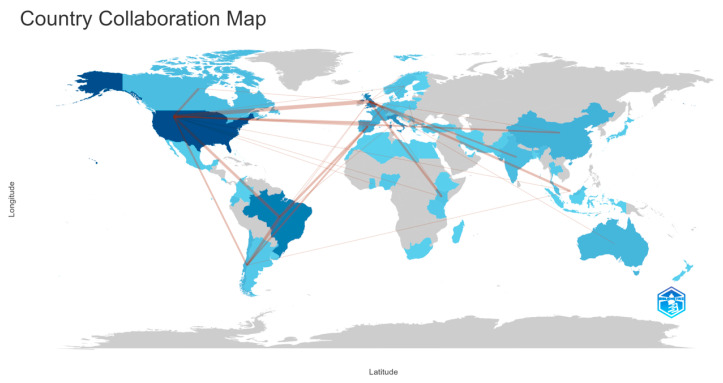
The Country Collaboration Map depicts international research collaborations in One Health studies related to coastal and marine contexts. Countries are shaded according to their publication volume, with darker blue tones indicating a higher number of documents, while grey represents countries with no publications. Connecting lines indicate the strength and direction of collaborative ties based on co-authorship, revealing concentrated research networks among high-income countries and limited contributions from low- and middle-income regions.

The Country Collaboration Map (Figure 11) illustrates the global distribution and intensity of research partnerships in OH studies applied to coastal and marine contexts. The darker shades indicate higher national research output, with the United States, several European countries, Brazil, and China emerging as the most prolific contributors. The connecting lines show co-authorship and international collaboration networks, highlighting strong transatlantic and intra-European ties as well as active linkages between North and South America. By contrast, many regions in Africa, the Middle East, and parts of Southeast Asia remain underrepresented, underscoring the geographic imbalances in research participation and the limited engagement of low- and middle-income coastal countries despite their high vulnerability to climate and health risks.

## 4. Discussion

The results of this bibliometric analysis reveal both the rapid expansion and the uneven development of One Health (OH) research in coastal and marine ecosystems. Annual scientific output remained minimal and inconsistent until 2018 but accelerated sharply after 2020, reaching a peak of 37 publications in 2024 (Figure 2). This shift illustrates the growing relevance of OH in addressing the complex interplay of ecosystem degradation, antimicrobial resistance (AMR), and climate-driven risks in coastal settings [32,33].

The keyword co-occurrence network (Figure 3) identified two major clusters: one focusing on OH, human, nonhuman, animals, and environmental health, and another dominated by antibiotic resistance, microbial genetics, and veterinary or medical applications [40,56]. This partial separation underscores the persistence of disciplinary silos, with limited integration across biomedical and environmental perspectives [1].

Thematic mapping (Figure 4) further clarified the conceptual structure of the field. Climate change, antibiotic resistance, epidemiology, and environmental monitoring emerged as motor themes, indicating mature and influential areas of research [17,68]. In contrast, governance approaches and food safety were positioned as transversal themes, while topics such as pathogen transmission, oil spills, and conceptual frameworks appeared as emerging or declining areas [74]. These findings highlight both the consolidation of core OH topics and the underdevelopment of cross-sectoral indicators such as chlorophyll-a or microplastics, despite their recognized policy significance [24,29].

Publication patterns also demonstrate concentration. According to Bradford’s Law (Figure 6), a small number of journals, including *One Health*, *Science of the Total Environment*, and *Pathogens*, act as the core outlets, while the majority of papers are dispersed across a long tail of less productive journals. Similarly, the distribution of authorship (Figure 7) follows Lotka’s Law, with most researchers contributing a single paper and only a handful publishing recurrently, confirming the emerging but still fragmented nature of this research domain [44].

Geographically, the analysis shows dominance by the United States, Brazil, and the United Kingdom (Figure 8 and Figure 9). Keyword usage in these countries emphasizes AMR and integrative OH themes, while contributions from low- and middle-income coastal regions remain limited, despite their high vulnerability to climate and health risks [10,11]. This imbalance is further reflected in the Country Collaboration Map (Figure 11), which highlights strong networks among high-income countries but weaker engagement in other regions [75,76,77].

The MCA (Figure 10) illustrates a clear dual orientation: a biomedical cluster focusing on antibiotics, resistance mechanisms, and laboratory studies, and an environmental cluster centered on microplastics, heavy metals, and estuarine ecosystems. The spatial separation between these clusters demonstrates limited integration but also signals opportunities for convergence between ecological and biomedical OH perspectives [1,59].

## 5. Conclusions

This bibliometric review mapped the application of the One Health (OH) framework in coastal and marine contexts over the past two decades. The analysis showed that scientific production remained sporadic until 2018 but expanded rapidly after 2020, peaking at 37 publications in 2024, confirming the growing relevance of OH in addressing complex socio-ecological challenges in vulnerable coastal zones.

Keyword networks and thematic mapping revealed two distinct but partially connected clusters: one oriented toward microbiology and antimicrobial resistance (AMR), and another focused on environmental and public health themes. While aquaculture disease management and pathogen surveillance are well-established topics, cross-cutting issues such as climate–health linkages, microplastics, and integrated ecological–health indicators (e.g., chlorophyll-a) remain underrepresented and need further development.

The analysis of sources and authors highlighted a small but interdisciplinary research community, with publications concentrated in a few key journals and groups. However, geographical distribution remains uneven, dominated by high-income countries, while low- and middle-income coastal regions, often most vulnerable to climate and health risks, are underrepresented. Collaboration patterns suggest limited but growing interdisciplinary integration, pointing to the need for stronger convergence of biomedical and ecological research agendas.

Overall, the findings demonstrate both the progress and the fragmentation of OH research in coastal and marine systems. To realize the full potential of the OH approach in this domain, future research should expand beyond high-income settings, enhance interdisciplinary collaboration, and systematically integrate cross-sectoral indicators into monitoring and governance frameworks. Such efforts will help bridge current thematic and geographical gaps, while reinforcing the role of OH in supporting coastal resilience and advancing the relevant SDGs.

## Figures and Tables

**Table 1:** Most cited articles in the field of One Health and environmental health, including author, year, journal, addressed theme, and total citation counts. The table complements Figure 2 by providing thematic insights into the topics driving scientific influence.

Author (Year)	Journal	Theme Addressed	Total Citations
[5]	The Lancet	Planetary Health: conceptual framework linking human health and global change	1947
[51]	Emerging Infectious Diseases	Emerging infectious diseases and zoonotic spillover patterns	228
[52]	Nature Reviews Cardiology	Climate change impacts on cardiovascular disease	190
[53]	Journal of the World Aquaculture Society	Aquaculture sustainability and food systems	98
[54]	PLOS ONE	Antimicrobial use in livestock and AMR risks	95
[55]	One Health	Operational frameworks and approaches for One Health	87
[56]	Pathogens	Epidemiology of zoonotic diseases (COVID-19 focus)	52
[57]	Reviews on Environmental Health	Environmental degradation and its impacts on health	49
[58]	Marine Pollution Bulletin	Marine pollution and human/ecosystem health	45

## Data Availability

No new data were created or analyzed in this study.
